# Using the Item‐Specific Deficit Approach to predict everyday functioning in mild cognitive impairment and Alzheimer's disease

**DOI:** 10.1002/alz70857_106229

**Published:** 2025-12-25

**Authors:** Jordan Kozuki, Elizabeth Matousek, Veronica Medrano‐Flores, Elena Rodriguez, Ellen Woo, Matthew J Wright

**Affiliations:** ^1^ California State University, Fresno, Fresno, CA, USA; ^2^ University of California, San Francisco ‐ Fresno, Fresno, CA, USA

## Abstract

**Background:**

Verbal memory impairment is a hallmark of Alzheimer's disease (AD) and can significantly impact activities of daily living (ADLs). Verbal memory includes the subprocesses of encoding, consolidation, and retrieval, which are typically assessed on list learning tasks using an overall performance score. The Item‐Specific Deficit Approach (ISDA) examines performances on individual test items, and this psychometrically sound method separates encoding, consolidation, and retrieval. The present study utilizes the ISDA to examine how these components of memory predict ADLs in mild cognitive impairment (MCI) and AD.

**Method:**

Participants included 58 healthy controls (HC), 65 persons with MCI, and 18 individuals with AD. To measure verbal memory, participants completed the California Verbal Learning Test‐II (CVLT‐II). The ISDA was applied to CVLT‐II scores to derive encoding, consolidation, and retrieval deficit indices. Everyday functioning was evaluated using the Everyday Cognition Scales (Ecog), which assesses cognitively mediated ADLs.

**Result:**

Hierarchal regressions were conducted to determine the roles of the ISDA indices (encoding, consolidation, and retrieval) in predicting ADLs. In MCI and AD, encoding and consolidation predicted everyday memory and everyday executive functioning: organization. In AD, encoding and consolidation additionally predicted everyday visual‐spatial abilities and total everyday cognition.

**Conclusion:**

The ISDA, which is a psychometrically sound method that separates encoding, consolidation and retrieval, is useful for predicting everyday functioning in those with MCI and AD. The ability to encode and consolidate information predicts everyday memory and executive functioning in those with mild cognitive difficulties. With more severe cognitive difficulties, such as AD, encoding and consolidation predict overall everyday function. These findings demonstrate the importance of assessing encoding, consolidation, and retrieval, not only for the purposes of diagnosis, but also when evaluating patients’ everyday functioning.

